# Fitness costs associated with acetyl‐coenzyme A carboxylase mutations endowing herbicide resistance in American sloughgrass (*Beckmannia syzigachne* Steud.)

**DOI:** 10.1002/ece3.4917

**Published:** 2019-01-28

**Authors:** Long Du, Mingjing Qu, Xiaojing Jiang, Xiao Li, Qian Ju, Xingtao Lu, Jinxin Wang

**Affiliations:** ^1^ Pest Bio‐control Lab Shandong Peanut Research Institute Qingdao China; ^2^ Institute of Plant Protection Tai'an Academy of Agricultural Sciences Tai'an China; ^3^ College of Plant Protection Shandong Agricultural University Tai'an China

**Keywords:** ACCase mutation, competition, evolution of resistance, fitness cost, plant growth, resistance

## Abstract

1. Weed resistance to herbicide can be conferred by gene mutations, and some mutations can cause pleiotropic effects in some cases. We investigated the pleiotropic effects associated with five specific ACCase mutations (Ile1781Leu, Trp2027Cys, Ile2041Asn, Asp2078Gly, and Gly2096Ala) on the plant growth, seed production, and resource competitiveness in American sloughgrass.
2. Resistant plants (M/M) homozygous for specific ACCase mutation and susceptible wild‐type plants (W/W) were derived from single heterozygous mother plant (M/W) by genotyping. Plant growth assay and neighborhood experiments were performed to quantify variation between M/M plants and W/W plants.
3. The Ile1781Leu mutation resulted in slight increases in plant growth in pure stands and improved resource competitiveness under low‐competition conditions in pot experiments, but no clear variation was observed under high competitive pressure or field conditions. During competition with wheat plants under field conditions, American sloughgrass plants containing Ile2041Asn ACCase exhibited a significantly lower (12.5%) aboveground biomass but no distinct differences in seed production or resource competitiveness. No significant detrimental pleiotropic effects associated with Gly2096Ala were detected in American sloughgrass.
4. The Trp2027Cys mutation distinctly reduced seed production, especially under high competitive pressure, but did not significantly alter plant growth. The Asp2078Gly mutation consistently reduced not only plant growth and seed production but also resource competitiveness.
5. *Synthesis*. The Trp2027Cys and Asp2078Gly mutations led to significant fitness costs, which may reduce the frequency of resistance alleles and reduce the propagation speed of resistant weeds in the absence of ACCase inhibitor herbicides. The Ile1781Leu, Ile2041Asn, and Gly2096Ala mutations displayed no obvious fitness costs or displayed very small fitness penalties, which would likely have no effect on the establishment of resistant weeds in the field.

## 1 INTRODUCTION

Acetyl‐coenzyme A carboxylase (ACCase, EC 6.4.1.2) is a biotinylated enzyme that catalyzes the carboxylation of acetyl‐CoA to produce malonyl‐CoA (Délye, Zhang, Michel, Matejicek, & Powles, 2005). ACCase is the common target site of three ACCase‐inhibiting herbicide chemistries: aryloxyphenoxypropionates (APPs), cyclohexanediones (CHDs), and the more recent phenylpyrazolin class herbicide pinoxaden (DEN). The wide and persistent use of ACCase‐inhibiting herbicides has made many grass weeds evolved ACCase herbicide resistance as a result of ACCase mutations. To date, 13 distinct amino acid substitutions located in the CT domain of the plastidic ACCase gene have been identified in field‐evolved resistant weed biotypes: Ile1781Leu, Trp1999Cys, Trp2027Cys, Ile2041Asn, Ile2041Val, Asp2078Gly, Cys2088Arg, Gly2096Ala (Powles & Yu, 2010), Ile1781Val (Collavo, Panozzo, Lucchesi, Scarabel, & Sattin, 2011), Ile1781Thr (Kaundun, Hutchings, Dale, & McIndoe, 2013),Trp1999Leu (Scarabel, Panozzo, Varotto, & Sattin, 2011), Trp1999Ser (Kaundun, Bailly, Dale, Hutchings, & McIndoe, 2013), and Gly2096Ser (Beckie, Warwick, & Sauder, 2017).

Certain mutations that provide target site‐based herbicide resistance can negatively impact plant growth and fitness, especially when stress factors are removed from the environment (Délye, Jasieniuk, & Corre, 2013; Vila‐Aiub, Gundel, & Preston, 2015; Vila‐Aiub, Neve, & Powles, 2009b). Direct fitness costs associated with mutations endowing herbicide resistance have been strongly confirmed in artificially created *Arabidopsis thaliana* mutants that have a homogeneous genetic background (Roux, Gasquez, & Reboud, 2004). Several studies have also shown that field‐evolved resistance alleles can cause significant fitness costs under accurate genetic background control. For example, acetohydroxyacid synthase (AHAS) Pro197Arg mutations result in slower growth rates for rigid ryegrass (*Lolium rigidum* Gaud.) (Yu, Han, Vila‐Aiub, & Powles, 2010); homozygous Asp2078Gly ACCase black‐grass (*Alopecurus myosuroides*) exhibits significantly reduced biomass, height, and seed production (Menchari, Chauvel, Darmency, & Délye, 2008); and, compared with wild‐type seeds, Ile2041Asn ACCase Asia minor bluegrass (*Polypogon fugax*) seeds exhibit lower seed germination (Tang, Xu, Shen, & Chen, 2017). However, these resistance‐associated fitness costs are usually difficult to predict, and their expression depends on weed species and particular mutant alleles. For example, Ile1781Leu ACCase improves plant growth in foxtail millet (*Setaria italica*) (Wang, Picard, Tian, & Darmency, 2010) but not in *A. myosuroides*(Menchari et al., 2008).

Herbicide‐resistant and herbicide‐susceptible weeds from different geographical locations will probably exhibit genetic variability associated with non‐resistance fitness traits. Thus, to evaluate the fitness variation precisely, resistant and susceptible individuals should share a common genetic background except for the alleles endowing herbicide resistance. (Bergelson & Purrington, 1996; Cousens & Fournier‐Level, 2018; Papapanagiotou, Paresidou, Kaloumenos, & Eleftherohorinos, 2015; Vila‐Aiub et al., 2009b). For example, by using PCR‐based marker analysis, several researchers have generated segregating homozygous mutant and wild‐type ACCase black‐grass plants derived from heterozygous mutant plants (Délye, Menchari, Michel, Cadet, & Corre, 2013; Du et al., 2016; Menchari et al., 2008).

**Figure 1:**
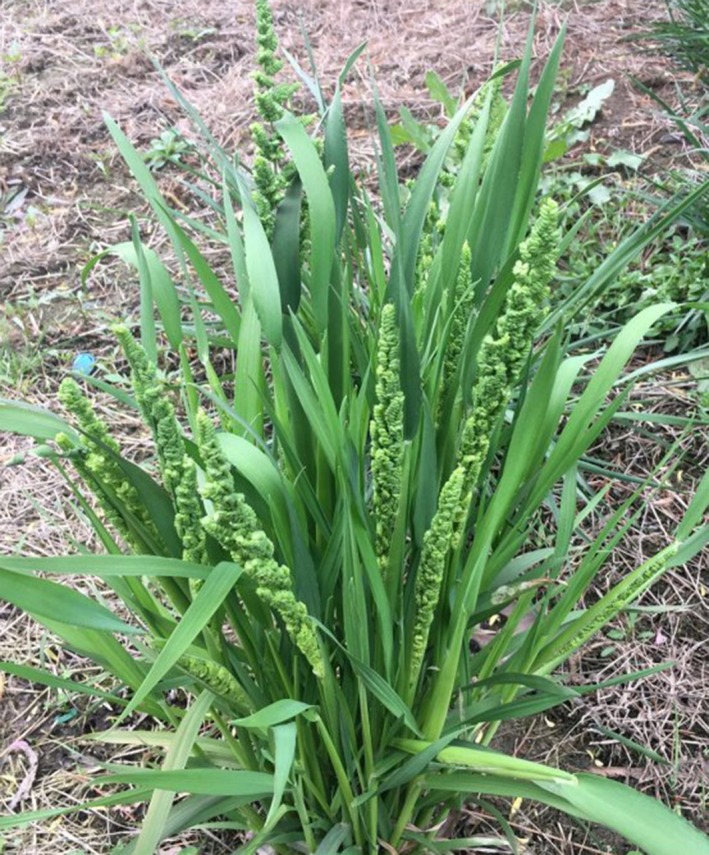
*Beckmannia syzigachne*, a member of the Poaceae family, is widely spread around the world including American, Europe, and Asia. Many *B. syzigachne* populations have evolved ACCase inhibitor resistance in southern China (Photographed by Wu minghua)

American sloughgrass (*Beckmannia syzigachne* Steud.), a member of the Poaceae family, is a widespread and severely harmful weed in wheat (*Triticum aestivum* L.) and oilseed rape (*Brassica napus* L.) fields in southern China. Many American sloughgrass populations have evolved ACCase inhibitor resistance, and various ACCase mutations have been identified in those resistant populations (Li, Du, Liu, Yuan, & Wang, 2014; Pan et al., 2015; Tang, Zhou, Zhang, & Chen, 2015). However, the effects of those ACCase mutations on the fitness‐related growth, seed production, and resource competitiveness of American sloughgrass have never been evaluated. In this study, to compare precisely the variation in fitness of resistant plants versus susceptible plants sharing a common genetic background, pairwise segregating resistant and susceptible progenies were generated from each original field population; each resistant genotype progeny was individually homozygous for the Ile1781Leu, Trp2027Cys, Ile2041Asn, Asp2078Gly, or Gly2096Ala mutation. The pleiotropic effects of those ACCase mutations on the fitness of this weed were subsequently assessed (Figure 1).

## 2 MATERIALS AND METHODS

### 2.1 Plant materials

Four field‐evolved resistant American sloughgrass populations were collected at different locations in southern China and were analyzed by ACCase genotyping and sequencing. Five ACCase mutations Ile1781Leu, Trp2027Cys, Ile2041Asn, Asp2078Gly, and Gly2096Al were observed. Two segregating genotype progenies were derived from a single mother plant that contained the specific heterozygous ACCase (M/W) mutation and no other known ACCase mutation. For each mutation, pairwise comparisons were performed between each M/M progenies and its corresponding W/W ones; both two genotype progenies were derived from the same original progenitor and shared a common genetic background, except for ACCase mutation of interest. Each mother plant was cultivated appropriately and isolated within a pollen‐proof enclosure during the flowering phase. After they ripened, F1‐progeny seeds from each mother plant were collected. Ten homozygous mutant (M/M) and ten wild‐type (W/W) ACCase plants in the F1 progeny were identified, and each group of ten mother plants was cultivated within a pollen‐proof enclosure to produce F2 seeds that were used for subsequent experiments (Table 1). And those progenies were the same ones used to assess cross‐resistance patterns associated with homozygous ACCase mutant alleles (Du et al., 2016). Ten plants of each F2 segregating progenies were randomly selected for ACCase sequencing, and all plants were confirmed to be homozygous for the specific mutant or wild‐type allele.

**Table 1:** Segregating progenies of each American sloughgrass genotype

Original population	Location	Mutational pattern	Derived segregating progenies
JS‐04	Jiangsu Danyang	Ile1781Leu	I/I1781‐JS‐04 (1781W/W) L/L1781‐JS‐04 (1781M/M)
AH‐12	Anhui Lujiang	Trp2027Cys	W/W2027‐AH‐12 (2027W/W) C/C2027‐AH‐12 (2027M/M)
JS‐32	Jiangsu Jintan	Ile2041Asn	I/I2041‐JS‐32 (2041W/W) N/N2041‐JS‐32 (2041M/M)
		Asp2078Gly	D/D2078‐JS‐32 (2078W/W) G/G2078‐JS‐32 (2078M/M)
SD*‐*04	Shandong Yutai	Gly2096Ala	G/G2096‐SD‐04 (2096W/W) A/A2096‐SD‐04(2096M/M)

### 2.2 Seed germination and growth

The seeds of the segregating F2 progenies were germinated in 9‐cm Petri dishes that contained two layers of filter paper soaked with 5 ml of distilled water; germination occurred in a growth chamber under a constant temperature of 10°C and a 12‐hr photoperiod (11,000 lx). After germination, the 1‐cm‐long seedlings were transplanted to plastic pots containing a mix of 50% sandy loam and 50% nursing media.

### 2.3 Assessment of plant growth

The relative growth rate (RGR) and its net assimilation rate (NAR) and leaf area ratio (LAR) components were analyzed for the homozygous mutant and wild‐type plants derived from the same single original population. The evaluation of growth traits involved classic and combined growth analyses (Hunt, 1982; Poorter, 1989).

With respect to the classic growth analysis experiments, uniform‐size seedlings from each genotype progeny were transplanted to individual plastic pots (20.5 cm diameter × 40 cm height) that contained the abovementioned substrate, after which the seedlings were grown in a greenhouse under day/night temperatures of 25/15 ± 5°C and natural sunlight. The plants were regularly watered and rearranged to randomize environmental differences within the greenhouse. The aboveground biomass of 10 plants from each progeny was collected for each date at 30 days and 50 days after the seedlings were transplanted. The leaf area was measured with a leaf area meter (Yaxin‐1242, Beijing Ya Xin Li Technology Co., Ltd., Beijing), after which the plants were harvested and then dried at 82°C for 3 days. The photosynthetic rates (µmol CO_2_ m^−2^ s^−1^) were estimated at 30 days and 50 days before the plants were cut (CIRAS‐3, PP Systems, Amesbury, MA). Photosynthesis was measured at a CO_2_ density of 400–410 μmol/mol under natural light at 25°C.

With respect to the combined growth analysis experiments, uniform‐size seedlings from each progeny were transplanted to plastic pots (40 pots for each progeny, 5 plants per pot, 20.5 cm diameter × 40 cm height) and cultivated under the abovementioned conditions. The leaf area and aboveground biomass were estimated at 25, 29, 33, 37, 41, 45, 49, 53, and 57 days after the seedlings were transplanted. Four pots (20 plants) were selected per harvest.

### 2.4 Assessment of resource competitiveness against wheat (reproductive stage) in pots

A plant's response to environmental resources is related to its ability to persist regardless of competitor presence. A neighborhood design was used to compare the reproductive biomass between mutant and susceptible plants derived from the same field population (Li, Yu, Han, Vila‐Aiub, & Powles, 2013; Vila‐Aiub, Neve, & Powles, 2009a). Four American sloughgrass plants per pot were subjected to competition from increasing densities of neighboring wheat plants (0, 30, 60, 120, 240, and 480 plants per m^2^). As such, germinated American sloughgrass and wheat seeds were simultaneously transplanted to pots and cultivated outdoors during the grown season. The pots were arranged in a completely randomized block design and were rearranged regularly. Before the wheat plants were harvested, the aboveground biomass and seeds of American sloughgrass and wheat plants were collected; each treatment consisted of four replicates.

### 2.5 Assessment of resource competitiveness against wheat in the field

The seeds of each genotype progeny were germinated as described above. The seedlings were transplanted to multipot plates and grown in a glasshouse. When the seedlings grew to 3 cm, forty uniform‐size plants (10 plants per block × 4 replicates) from each progeny were transplanted between wheat rows (17 cm apart) and spaced 30 cm apart within each row. Cultural practices in line with local conventional practices were performed, with the exceptions that no herbicides were applied during the whole grown season and that other grassy weeds were removed by hand. After the American sloughgrass plants matured, their aboveground biomass and seeds were collected. Four replicates were included per treatment in a fully randomized design.

### 2.6 Statistical analysis and regression analysis

To assess plant growth assessment, the RGR and its components were determined using an unbiased formula. The variance of the RGR was evaluated in accordance with the following formula:

(1) $$V\left(\text{RGR}\right)=\frac{1}{W}\cdot \frac{\text{d}w}{\text{d}t}=\frac{\text{ln}w_{2}-\text{ln}w_{1}}{t_{2}-t_{1}}$$

where *w*_1_ and *w*_2_ represent the plant dry weight at harvest times *t*_1_ and *t*_2_. One‐way analysis of variance (ANOVA) in conjunction with Tukey's honestly significant difference (HSD) test (*α* = 0.05) was performed to compare RGR and photosynthesis estimates between the mutant and susceptible progenies derived from the same original field population.

The combined growth analysis was performed using a splined cubic polynomial model to describe the comparison of time trends in the classically derived RGR, NAR, and LAR (Hunt & Evans, 1980; Poorter, 1989) as follows:

(2) $$y=y_{0}+ax+bx^{2}+cx^{3}$$

where *y* is the RGR, NAR, or LAR of the plant; *x* is the time; *y*_0_ is the *y* value when *x* = 0; and *a*, *b*, and *c* are the rates of increase at different times.

The per unit size competitiveness of each progeny against wheat in pots was analyzed using a hyperbolic non‐linear model (Vila‐Aiub et al., 2009a) as follows:

(3) $$y=\frac{a}{1+bx}$$

where *y* represents the seed production of American sloughgrass at wheat density or biomass *x*, *a* denotes the seed production of American sloughgrass in the absence of wheat (*x* = 0), and *b* is the slope of the regression. Steep slopes denote weak resource competitiveness.

In the field experiments, differences in plant height, aboveground biomass, and seed production between the mutant and susceptible genotypes of each group were compared using Tukey's HSD test (*α* = 0.05).

## 3 RESULTS

### 3.1 Assessment of plant growth

The results of the classic growth analysis during a 20‐day period (30–50 days after transplanting (DAT)) are shown in Table 2. The results of the pairwise comparisons between the homozygous mutant and wild‐type plants derived from the same population showed that the 1781M/M plants displayed greater RGR and NAR values than did the 1781W/W plants (increases of 10.5% and 31.7%, respectively), but the 1781M/M and 1781W/W plants exhibited similar photosynthetic rates (11.2–12.9 μmol CO_2_ m^−2^ s^−1^). Compared with those of the 2078W/W plants, the RGR and NAR of the 2078M/M plants decreased by 10.7% and 15.4%, respectively. No significant differences in classic growth traits were observed between the 2027M/M, 2041M/M, and 2096M/M plants and their respective corresponding 2027W/W, 2041W/W, and 2096W/W plants.

**Table 2:** Plant growth traits of each American sloughgrass genotype

Genotype	Growth traits			Photosynthesis (μmol CO_2_ m^−2^ s^−1^)	
	RGR (mg mg^−1^ days^−1^)	NAR (mg cm^−2^ days^−1^)	LAR (cm^2^/mg)	30 DAT	50 DAT
1781M/M	0.116a	0.237a	0.488b	12.9a	11.2a
1781W/W	0.105b	0.180b	0.586a	12.6a	11.5a
2027M/M	0.114a	0.218a	0.523a	11.5a	10.4a
2027W/W	0.109a	0.196b	0.557a	11.8a	10.3a
2041M/M	0.109a	0.219a	0.498a	11.3a	10.6a
2041W/W	0.111a	0.217a	0.513a	11.9a	10.2a
2078M/M	0.100a	0.186b	0.540a	11.2a	10.1a
2078W/W	0.112b	0.220a	0.510a	10.8a	9.8a
2096M/M	0.113a	0.220a	0.515a	12.7a	11.3a
2096W/W	0.111a	0.227a	0.489a	12.2a	11.1a

Note

DAT: days after transplanting the seedlings.

Significant differences were analyzed between the M/M and W/W plants derived from the same population (i.e., 1781M/M vs. 1781W/W) according to Tukey's HSD test (*α *= 0.05).

**Figure 2:**
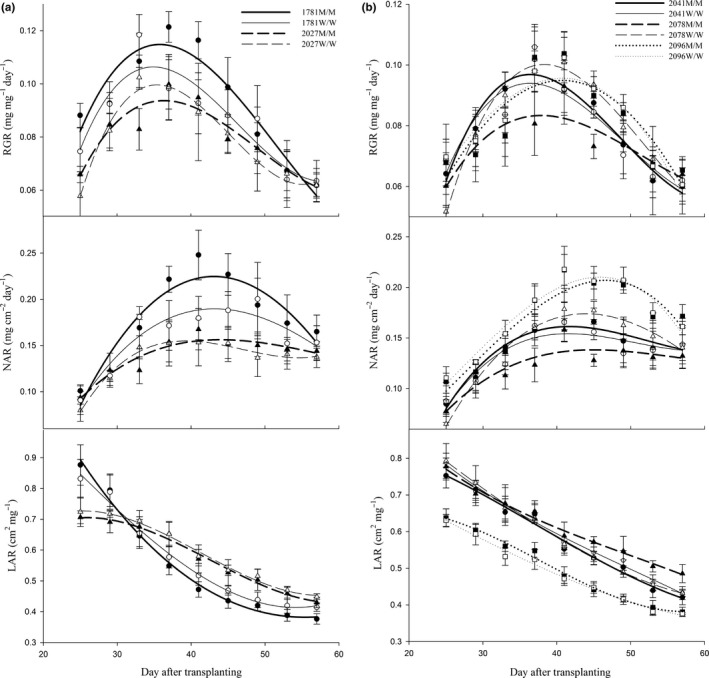
Changes in the mean estimates of the relative growth rate (RGR), net assimilation rate (NAR), and LAR over time for each American sloughgrass genotype

The combined growth analysis involved a longer time period that commenced 25 days post‐transplanting (25–57 DAT). The aboveground biomass and leaf area of the plants were measured every 4 days, after which the RGR, NAR, and LAR were calculated. The dynamic changes in the growth parameters of the plants of each genotype are shown in Figure 2a,b. During this growth period, the RGR and NAR values of all plants peaked at 34–37 and 41–45 DAT, respectively, but then decreased as time progressed. Population effects on growth traits were clearly observed between derived progenies.

The results of pairwise comparisons between mutant genotype plants and their corresponding wild‐type plants showed that the RGR and NAR time trends of the 1781M/M and 2078M/M plants significantly differed from those of the 1781W/W and 2078W/W plants, respectively. During this growth period, the increases in the RGR and NAR of the 1781M/M plants were 7.34% and 12.8%, respectively. In contrast, compared with those of the 2078W/W plants, the RGR (−8.53%) and NAR (−13.4%) of the 2078M/M plants derived from the same original population were clearly lower. However, the growth traits did not significantly differ between the 2027M/M, 2041M/M, and 2096M/M plants and their corresponding wild‐type plants (Figure 2a,b).

### 3.2 Assessment of resource competitiveness against wheat in pots

**Figure 3:**
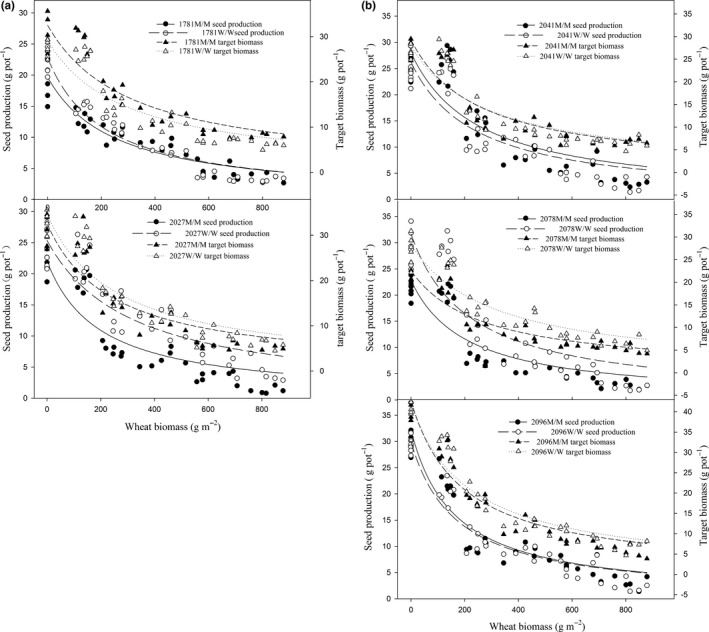
Aboveground biomass and seed production responses of each American sloughgrass genotype to the increasing aboveground vegetative biomass of wheat plants

The responses of each mutant and corresponding wild‐type target plant to resource pressure under increasing wheat competition were compared. Target American sloughgrass seed production and aboveground biomass were significantly affected by the increased density and biomass of competing wheat plants. The seed production per pot (four target plants) decreased in a similar manner from 20–30 g (control) to 13–25 g (30 plants per m^2^), 8–14 g (60 plants per m^2^), 6–9 g (120 plants per m^2^), 4–6 g (240 plants per m^2^), and finally 2–4 g (480 plants per m^2^) under increasing wheat density and biomass, and the aboveground biomass per pot decreased from 20–40 g (control) to 5–7 g (480 plants per m^2^). The results of comparisons between several wild‐type plants derived from different original field populations showed significant population effects on resource competitiveness to wheat. The 2096W/W plants accumulated more aboveground biomass than did the 1781W/W, 2027W/W, 2041W/W, and 2078W/W plants in the presence and absence of wheat competition (Figure 3a,b).

The results of pairwise comparisons between the mutant plants and their corresponding wild‐type plants derived from the same field population showed that different mutant patterns could result in pleiotropic effects on plant resource competitiveness. Compared with the 1781W/W plants, the 1781M/M plants exhibited slightly reduced seed production (12%–15%) and significantly increased (11%–18%) aboveground biomass under low competitive pressure (Figure 3a, Supporting Information Tables S1 and S2). Compared with that of the 2027W/W plants, the seed production of the 2027M/M plants decreased by 13.3%, 9.1%, 44.7%, 47.7%, 47.6%, and 66.5% at wheat competitive densities of 0, 30, 60, 120, 240, and 480 plants m^2^, respectively, (Figure 3a, Supporting Information Table S1). Compared with the 2027W/W plants, the 2027M/M plants exhibited a slight reduction in aboveground biomass. In the progenies segregating for Gly‐2078 ACCase, when grown in the presence of competition from increasing wheat density and biomass, the 2078M/M plants showed a weaker mean competitive response than did the 2078W/W plants. Compared with the 2078W/W plants, the 2078M/M plants produced less aboveground biomass (decreased by 18%–40%) and exhibited less seed production (decreased by 23%–41%) (Figure 3a,b, Supporting Information Tables S1 and S2). Compared with those of their corresponding 2041W/W and 2096W/W plants, the respective seed production and aboveground biomass of the 2041M/M and 2096M/M plants did not significantly vary (Figure 3b).

### 3.3 Assessment of resource competitiveness against wheat in the field

**Figure 4:**
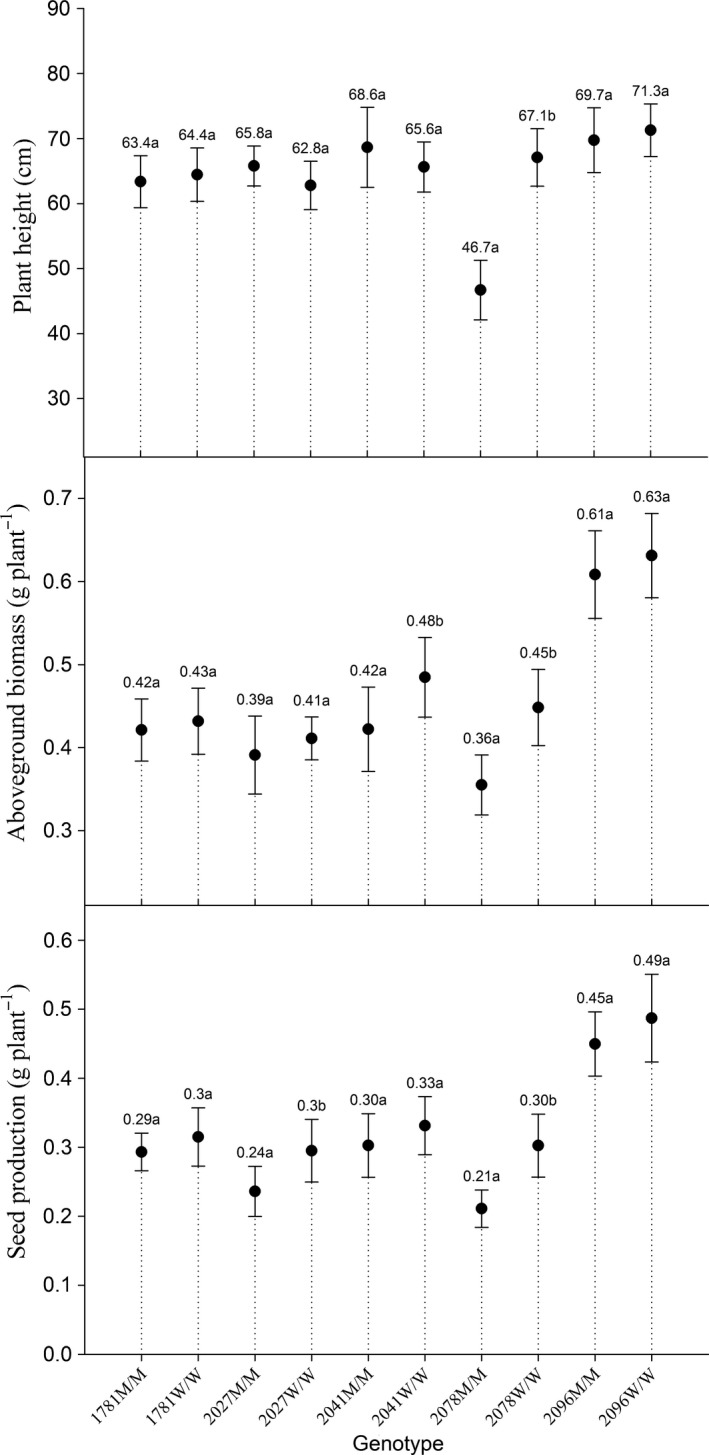
Comparisons of plant height, biomass, and seed production among each genotype. Significant differences were calculated between mutant genotypes and their corresponding wild‐type genotypes

To simulate a realistic growth environment, plants from ten derived genotype progenies were transplanted to the field to compete with wheat plants. The plant height, biomass, seed production, and number of tillers of each progeny were measured. Due to the competition with wheat, the effective number of tillers of most plants ranged from 1 to 2, so the tiller numbers of each genotype were not compared. The significant differences in plant height, biomass, and seed production of each genotype are shown in Figure 4.

As the field experiment conditions were worse than potting experiment conditions, the American sloughgrass biomass and seed production were lower under field conditions than under potting conditions. When comparing among the five wild‐type genotypes, the 2096W/W plants exhibited significantly greater plant height, biomass, and seed production than did the 1781W/W, 2027W/W, 2041W/W, and 2078W/W plants. The 2096W/W plants and the other four wild‐type plants were derived from different field populations; therefore, population effects could possibly have led to this variation (Table 3). The results of pairwise comparisons between each mutant plant and its corresponding wild‐type plant showed that, compared with that of the 2027W/W plants, the seed production in the 2027M/M plants decreased by approximately 20%, but plant height and aboveground biomass did not significantly vary. The 2078M/M plants displayed more obvious differences than did the wild‐type 2078W/W plants: Compared with the 2078W/W plants, the 2078M/M plants exhibited significant reductions in plant height (30%), aboveground biomass (20%), and seed mass (30%) (Figure 4). The biomass of the 2041M/M plants was 12.5% lower than that of the 2041W/W plants, but no significant differences in seed production or plant height were observed (Figure 4).

**Table 3:** Comparisons of plant height, biomass, and seed production among each wild‐type American sloughgrass genotype in the field experiment

Genotype	Plant height (cm)	Biomass (g)	Seed production (g)
1781W/W	64.4 (1.37)b	0.43 (0.013)bc	0.32 (0.014)b
2027W/W	62.8 (1.24)b	0.41 (0.009)c	0.29 (0.015)b
2041W/W	65.6 (1.28)b	0.48 (0.016)b	0.33 (0.014)b
2078W/W	67.1 (1.47)ab	0.45 (0.015)bc	0.30 (0.015)b
2096W/W	71.3 (1.35)a	0.63 (0.017)a	0.49 (0.021)a

Note

Significant differences were analyzed according to Tukey's HSD test (*α* = 0.05).

## 4 DISCUSSION

### 4.1 Controlling the genetic background

Because of various environmental pressures, different species ecotypes may evolve in different geographical locations. Different ecotype populations can exhibit genetic variability, which can result in fitness variation. Thus, differences between herbicide‐resistant and herbicide‐susceptible populations probably result from other non‐resistance‐associated fitness traits (Vila‐Aiub et al., 2009b). Therefore, to accurately assess herbicide resistance‐associated fitness costs, comparisons should be conducted between herbicide‐resistant plants and herbicide‐susceptible plants sharing a common genetic background. In this study, plants homozygous for specific ACCase mutations (M/M) and their corresponding wild‐type (W/W) plants were derived from a single mother plant heterozygous for the specific ACCase mutation. This approach ensured that resistant and susceptible plants shared a common genetic background, with the exception of the mutant allele providing herbicide resistance.

### 4.2 Comparison of plant growth

Herbicide resistance endowed by target site mutations in weeds often adversely affects their growth and fitness (Vila‐Aiub et al., 2009b; Yu et al., 2010). This negative influence may be due to a mutant gene that alters the target enzyme catalytic activity; the mutant gene either reduces the affinity of the target enzyme for its substrate or alters the feedback inhibition of the target enzyme, which in turn causes the enzyme to catalyze the synthesis of its product in excessive or inadequate amounts. In resistant *L. rigidum* plants whose resistance is conferred by ACCase mutations, the ACCase Asp2078Gly mutation can lead to decreases in the RGR and the NAR by 30% and 38%, respectively, during the vegetative growth stage. It is reasonable that the degradation of ACCase activity and the variation in enzyme kinetic parameters result in these pleiotropic effects on plant growth (Vila‐Aiub, Yu, Han, & Powles, 2015). However, unlike the changes in enzyme functionality and plant growth caused by mutations in target genes, many target gene mutations do not lead to those changes. Also, in resistant *L. rigidum* plants whose resistance is conferred by ACCase mutations, the ACCase Ile1781Leu mutation resulted in enzyme activity, enzyme kinetic parameters, RGR, aboveground and belowground biomass, leaf area, and other parameters that were not significantly differ from those of wild‐type *L. rigidum*.

In this study, the classical growth assay results showed that the homozygous ACCase Ile1781Leu mutation could increase the American sloughgrass RGR and NAR by 10.5% and 31.7%, respectively, which indicated a negative effect on plant growth. But in some case of other ACCase‐resistant weeds, no significant effects on plant growth were observed such as *L. rigidum* (Vila‐Aiub, Neve, & Powles, 2005; Vila‐Aiub, Yu et al., 2015) and *A. myosuroides* (Menchari et al., 2008). In addition, the ACCase Ile1781Leu mutation in *S. italica* (L.) Beauv. could lead to better and earlier plant growth in the absence of herbicide selection (Wang et al., 2010). It is plausible that the effect of gene mutations on weed growth varies depending on the weed species; however, American sloughgrass plants homozygous for the Asp2078Gly mutation displayed significant reductions in RGR (10.7%) and NAR (15.4%). These results are in agreement with those of most studies, indicating growth penalties associated with the Asp2078Gly mutation occurred (Vila‐Aiub, Yu et al., 2015). Unlike the Ile1781Leu and Asp2078Gly mutations, the homozygous ACCase Trp2027Cys, Ile2041Asn, and Gly2096Ala mutations did not affect the growth of American sloughgrass. The photosynthetic rate can directly affect plant growth. However, the photosynthetic rate measurements indicated that all genotypes exhibited similar photosynthetic rate time trends. The photosynthetic rate of weeds is inconsistent with their growth rate, as reported for *Raphanus raphanistrum*(Li et al., 2013) and *Avena fatua*(Lehnhoff, Keith, Dyer, Peterson, & Menalled, 2013). In this study, the photosynthetic rate contributed little to the variation in fitness of resistant American sloughgrass plants.

### 4.3 Estimation of ecological fitness

It is generally known that environmental factors can strongly affect plant growth (Yu, Ahmad‐Hamdani, Han, Christoffers, & Powles, 2013), and the ecological fitness costs of plants may be more evident when plants are growing under stressful environmental conditions (Yang, Dong, Li, & Moss, 2007), such as predation (Gassmann, 2005), disease (Brown, 2003), and/or resource competition. If the ability of herbicide‐resistant weeds to acquire resources diminishes, ecological fitness costs may be more readily discovered under conditions of intense resource competition. For instance, *A. thaliana* plants containing the ALS Pro197Ser mutation exhibited a significant decrease (31%) in seed production under field conditions in the absence of fertilization. However, this variation was not detected under sufficient fertilizer conditions (Purrington & Bergelson, 1997).

In this study, pot and field experiments were conducted to evaluate the variation in ecological fitness between resistant and susceptible weeds derived from the same field population. The American sloughgrass biomass and seed production decreased significantly as the density of neighboring wheat plants increased. Compared with corresponding wild‐type plants, American sloughgrass plants containing Ile1781Leu ACCase exhibited increased (10.9%–18.1%) biomass but reduced (approximately 10%) seed production under low‐competition conditions in the pot experiment, but no significant differences occurred under high competitive pressure. Therefore, it is difficult to accurately assess whether the competitive response is weaker or stronger. However, in the field experiment, no significant differences in seed production, plant height, or aboveground biomass were observed between the Ile1781Leu and wild‐type plants. The mutant Ile1781Leu ACCase seemingly causes no significant ecological fitness costs in American sloughgrass. This result is in agreement with those of most reports. However, the mutant Ile1781Leu ACCase in *S. italica* (L.) Beauv. improved plant fitness, increasing plant growth; the increase in the frequency of resistant plants in stressed F4 and F5 field populations is more accurate proof of this phenomenon (Wang et al., 2010). It is likely difficult to predict the fitness costs associated with resistance alleles on a case‐by‐case basis. Moreover, the pleiotropic effects associated with resistance alleles are multitudinous, so it is important to use a range of evaluations throughout the whole plant life cycle and under some competitive pressures to detect small variations in fitness.

Asp2078Gly ACCase generally results in fitness costs in plant growth and resource competitiveness (Menchari et al., 2008; Vila‐Aiub, Yu et al., 2015). Homozygous Asp2078Gly mutations could clearly reduce American sloughgrass biomass and seed production. This result is consistent with those of most reports. One explanation for fitness costs associated with Asp2078Gly is that this mutant allele may reduce enzyme activity (Vila‐Aiub, Yu et al., 2015). Impaired ACCase activity can cause a shortage of lipids available for plant growth and is correlated with impaired resource competitiveness in plants homozygous for the Asp2078Gly mutation (Vila‐Aiub, Yu et al., 2015).

To date, only one published study has evaluated the ecological fitness costs associated with the Trp2027Cys mutation in *Avena sterilis*(Papapanagiotou et al., 2015), and no definitive ecological fitness costs were detected. In our study, the homozygous Trp2027Cys mutation could lead to a significant decrease in seed production; in addition, as the wheat planting density increased, the decrease in seed production became more pronounced. When the density of the wheat plants was 480 plants per m^2^, the seed production decreased by 66.5%. In other words, plants containing Trp2027Cys ACCase showed a distinctly weaker competitive response to increasing wheat density, although no significant variation in plant growth was observed. In our previous study, the Trp2027Cys ACCase did not significantly affect seed germination (Du et al., 2017). Throughout the whole American sloughgrass growth period, fitness costs associated with the Trp2027Cys ACCase were detected only during the reproductive stage. This impaired trait may lead to an incompatibility with the natural ecosystem in the field and reduce the risk of spreading in cropping systems in the absence of herbicides.

We found that the Ile2041Asn mutation caused no obvious variation in responses to resource competition in the pot experiment. Interestingly, when growing under competition with wheat plants in the field, the plants with the Ile2041Asn allele exhibited reduced (12.5%) aboveground biomass, but no evident difference in seed production or plant height was observed in the field experiment. These results agree with similar results reported for *A. myosuroides* growing in competition with wheat plants in the field: The Ile2041Asn mutation caused no obvious fitness costs in terms of plant growth, seed production, and plant height (Menchari et al., 2008). In our study, no pleiotropic effects associated with Gly2096Ala ACCase were observed. Presumably, the Ile2041Asn or Gly2096Ala ACCase mutation had little effect on the reproduction of American sloughgrass in the field.

### 4.4 Management and evolution of ACCase target site herbicide resistance

Different ACCase mutations can cause different cross‐resistance spectrum. The results of our previous studies indicated that all five of these ACCase mutations can cause broad cross‐resistance to fenoxaprop‐*p*‐ethyl, clodinafop‐propargyl, fluazifop‐*p*‐butyl, haloxyfop‐*p*‐methyl, sethoxydim, clethodim, and pinoxaden in American sloughgrass (Du et al., 2016). This fact increases the difficulty of resistance management and compels growers to select other herbicides that have different mechanisms of action. Alternatively, fitness costs associated with specific ACCase mutations may provide growers a new weed control strategy. Cultural practices such as plowing, planting competitive crops, close planting, and planting cover crops could maximize the fitness penalty for some plants containing mutant forms of ACCase (Chauvel, Guillemin, Colbach, & Gasquez, 2001; Menchari et al., 2008). Fortunately, American sloughgrass plants containing Trp2027Cys or Asp2078Gly ACCase displayed significant fitness costs, especially under competitive pressure in the absence of ACCase inhibitor herbicides; therefore, the abovementioned cultural practices can be used as supplementary management tools for controlling those resistant alleles. The frequency of the Trp2027Cys and Asp2078Gly alleles is predicted to decrease in natural field ecosystems in which ACCase herbicides are not applied. However, the lack of significant detrimental effects on plant growth and competitiveness associated with the Ile1781Leu, Ile2041Asn, and Gly2096Ala mutations could contribute to the persistence of the resistance allele in populations that do not select for ACCase herbicides. To verify our inference, additional studies monitoring resistance gene frequencies for several years should be conducted in the field in the absence of ACCase herbicides.

In summary, to accurately estimate fitness costs associated with specific ACCase mutations conferring herbicide resistance to American sloughgrass, mutant plants (individually homozygous for the Ile1781Leu, Trp2027Cys, Ile2041Asn, Asp2078Gly, or Gly2096Ala mutation) and wild‐type plants were generated from single mother plants that were individually heterozygous for the specific ACCase mutations, and several key parameters of the life cycle of this grassy weed were measured, including those parameters under conditions of resource competition pressure. The Trp2027Cys and Asp2078Gly mutations led to significant fitness costs, which may reduce the frequency of resistance alleles and reduce the propagation speed of resistant weeds in the absence of ACCase inhibitor herbicides. The Ile1781Leu, Ile2041Asn, and Gly2096Ala mutations displayed no obvious fitness costs or displayed very small fitness penalties, which would likely have no effect on the establishment of resistant weeds in the field.

## CONFLICT OF INTEREST

The authors declare that the research was conducted in the absence of any commercial or financial relationships that could be considered as a conflict of interest.

## AUTHORS' CONTRIBUTIONS

LD, MQ, and JW designed the research. LD, MQ, and XJ performed the experimental work and the data analysis. QJ, XL, and XL provided helpful suggestion in data analysis and manuscript preparation. LD and JW supervised the research and manuscript preparation. All authors contributed critically to the drafts and gave final approval for publication.

## Data Availability

All authors agree with archiving our data in Dryad after acceptance.
