# Supplementary material for: Fitness costs associated with acetyl‐coenzyme A carboxylase mutations endowing herbicide resistance in American sloughgrass (Beckmannia syzigachne Steud.)
Source: Ecol Evol. 2019 Jan 28;9(4):2220–30. doi: 10.1002/ece3.4917 (PMC6392401; doi:10.1002/ece3.4917)
Supplement: Supplementary file 1 [file ECE3-9-2220-s001.docx]

Supporting Information

**Table S1** *Seed production of each American sloughgrass genotype along a density gradient of wheat plants*

| **Genotype** | **Seed production (g pot^-1^)** | | | | | |
| --- | --- | --- | --- | --- | --- | --- |
|  | **0**  **plant m^-2^** | **30**  **plants m^-2^** | **60**  **plants m^-2^** | **120**  **plants m^-2^** | **240**  **plants m^-2^** | **480**  **plants m^-2^** |
| 1781M/M | 19.58 **a** | 12.73 **a** | 10.55 **a** | 8.68 **a** | 4.83 **a** | 3.48 **a** |
| 1781W/W | 22.31 **a** | 14.96 **a** | 12.09 **a** | 7.87 **a** | 3.60 **a** | 3.30 **a** |
| 2027M/M | 20.64 **a** | 19.17 **a** | 7.78 **b** | 6.26 **b** | 3.65 **b** | 1.37 **b** |
| 2027W/W | 23.81 **a** | 21.68 **a** | 14.08 **a** | 11.97 **a** | 6.96 **a** | 4.09 **a** |
| 2041M/M | 25.87 **a** | 25.35 **a** | 14.72 **a** | 7.92 **a** | 6.21 **a** | 3.12 **a** |
| 2041W/W | 24.33 **a** | 24.04 **a** | 10.40 **b** | 9.58 **a** | 5.04 **a** | 2.81 **a** |
| 2078M/M | 21.27 **b** | 20.49 **b** | 7.59 **b** | 6.24 **a** | 4.47 **a** | 2.78 **a** |
| 2078W/W | 29.11 **a** | 29.15 **a** | 12.94 **a** | 8.11 **a** | 6.04 **a** | 2.43 **a** |
| 2096M/M | 31.37 **a** | 22.27 **a** | 9.82 **a** | 8.61 **a** | 6.58 **a** | 3.10 **a** |
| 2096W/W | 29.31 **a** | 20.20 **a** | 10.86 **a** | 8.75 **a** | 6.03 **a** | 2.48 **a** |

Note: Wheat density: 0, 30, 60, 120, 240, 480 plants m^-2^. Significant differences were analyzed between the M/M and W/W plants derived from the same population (i.e. 1781M/M VS 1781W/W) according to Tukey’s HSD test (α=0.05).

**Table S2** *Aboveground biomass of each American sloughgrass genotype along a density gradient of wheat plants*

| **Genotype** | **Above-ground biomass (g pot^-1^)** | | | | | |
| --- | --- | --- | --- | --- | --- | --- |
|  | **0**  **plant m-^2^** | **30**  **plants m^-2^** | **60**  **plants m^-2^** | **120**  **plants m^-2^** | **240**  **plants m^-2^** | **480**  **plants m^-2^** |
| 1781M/M | 30.09 **a** | 29.39 **a** | 17.62 **a** | 12.39 **a** | 8.21 **a** | 7.76 **a** |
| 1781W/W | 27.13 **a** | 26.13 **b** | 14.92 **b** | 10.73 **a** | 6.95 **a** | 6.30 **a** |
| 2027M/M | 30.65 **a** | 27.96 **a** | 15.27 **a** | 10.52 **a** | 5.64 **a** | 5.59 **a** |
| 2027W/W | 32.52 **a** | 29.84 **a** | 16.28 **a** | 12.27 **a** | 7.64 **a** | 6.33 **a** |
| 2041M/M | 27.69 **a** | 25.98 **a** | 11.33 **a** | 9.78 **a** | 6.81 **a** | 6.77 **a** |
| 2041W/W | 25.84 **a** | 25.91 **a** | 12.83 **a** | 9.26 **a** | 7.08 **a** | 6.44 **a** |
| 2078M/M | 21.35 **b** | 19.73 **b** | 8.90 **b** | 8.40 **b** | 5.88 **b** | 5.43 **a** |
| 2078W/W | 26.93 **a** | 24.03 **a** | 14.77 **a** | 11.58 **a** | 8.17 **a** | 6.37 **a** |
| 2096M/M | 41.50 **a** | 30.11 **a** | 18.25 **a** | 11.78 **a** | 7.90 **b** | 5.77 **a** |
| 2096W/W | 40.28 **a** | 31.82 **a** | 19.01 **a** | 11.69 **a** | 10.09 **a** | 6.69 **a** |

Note: Wheat density 0, 30, 60, 120, 240, 480 plants m^-2^. Significant differences were analyzed between the M/M and W/W plants derived from the same population (i.e. 1781M/M VS 1781W/W) according to Tukey’s HSD test (α=0.05).

**Table S3** *Comparisons of competitive responses (b parameter) between M/M and W/W phenotypes to wheat*

| **Genotype** | **Seed production** | | **Aboveground biomass** | |
| --- | --- | --- | --- | --- |
|  | **b** | ***R^2^*** | **b** | ***R^2^*** |
| 1781M/M | 0.0040 (0.0004) | 0.8937 | 0.0032 (0.0004) | 0.8434 |
| 1781W/W | 0.0047 (0.0004) | 0.9407 | 0.0034 (0.0004) | 0.8550 |
| 2027M/M | 0.0051 (0.0009) | 0.8139 | 0.0042 (0.0006) | 0.8435 |
| 2027W/W | 0.0031 (0.0005) | 0.8233 | 0.0038 (0.0005) | 0.8679 |
| 2041M/M | 0.0040 (0.0007) | 0.7934 | 0.0040 (0.0006) | 0.8350 |
| 2041W/W | 0.0042 (0.0007) | 0.7811 | 0.0036 (0.0005) | 0.8260 |
| 2078M/M | 0.0048 (0.0008) | 0.8036 | 0.0037 (0.0006) | 0.8048 |
| 2078W/W | 0.0046 (0.0009) | 0.7812 | 0.0032 (0.0004) | 0.8706 |
| 2096M/M | 0.0062 (0.0007) | 0.9086 | 0.0053 (0.0004) | 0.9409 |
| 2096W/W | 0.0059 (0.0006) | 0.9263 | 0.0046 (0.0004) | 0.9193 |

Note: Values represent the mean estimate of slopes (b parameter) derived from significant regressions y = a/(1 + bx). Values in parenthesis denote standard error of the mean.
